# META-ANALYSIS OF INTERVENTIONS TO REDUCE SEDENTARY BEHAVIOR AMONG OLDER ADULTS

**DOI:** 10.1093/geroni/igz038.3183

**Published:** 2019-11-08

**Authors:** Jennifer A Otmanowski, Sheri A Rowland, Pamela S Cooper, Jo-Ana D Chase

**Affiliations:** 1 University of Missouri-Columbia, Concord, Michigan, United States; 2 University of Nebraska Medical Center, Lincoln, Nebraska, United States; 3 Cooper Editorial and Scientific Consulting, Columbia, Missouri, United States; 4 University of Missouri, Columbia, Missouri, United States

## Abstract

ABSTRACT BODY Sedentary behavior (SB) is associated with substantial health risks such as increased risk of cardiovascular mortality, diabetes, and cognitive and physical functioning decline. Older adults are particularly at risk as they are the most sedentary population. The purpose of this meta-analysis was to determine the overall effects of interventions designed to reduce SB among older adults. A comprehensive literature search of online databases, bibliographies, and author searches located published and unpublished studies. Included studies tested interventions to reduce SB time, were written in English, and focused on community-dwelling adults age 60 years or older. Data were extracted on sample, study design, and intervention characteristics using an investigator-developed tool. Study effect sizes were synthesized using a random effects model. Heterogeneity of effects across studies was examined; however, moderator analyses were not conducted due to the small number of included studies. Of the 2,408 reviewed citations, 22 reports were included representing 17 distinct studies, eight of which were included in the two-group post-test meta-analysis (n= 1,024 participants). Interventions overall modestly reduced SB time among older adults (d=-.25, 95% CI [-.50, .00], p=.05); however, significant heterogeneity of effect size was observed across studies (Q=22.34, p<.01). Our findings demonstrate a need for more research targeting SB reduction in this high-risk population. Future research should include measures of breaks in sedentary time and types of SB (e.g., watching TV, reading) which are also critical indicators of health risk. Moreover, further exploration of relationships between health outcomes and SB intervention effects is needed.

